# Cholinergic System Under Aluminium Toxicity in Rat Brain

**DOI:** 10.4103/0971-6580.72682

**Published:** 2010

**Authors:** K. Yellamma, S. Saraswathamma, B. Nirmala Kumari

**Affiliations:** Department of Zoology, Sri Venkateswara University, Tirupati, Andhra Pradesh, India

**Keywords:** Aluminium acetate, behavioral changes, cholinergic system, rat brain

## Abstract

The present investigation envisages the toxic effects of aluminium on the cholinergic system of male albino rat brain. Aluminium toxicity (LD_50_/24 h) evaluated as per Probit method was found to be 700 mg/kg body weight. One-fifth of lethal dose was taken as the sublethal dose. For acute dose studies, rats were given a single lethal dose of aluminium acetate orally for one day only and for chronic dose studies, the rats were administered with sublethal dose of aluminium acetate once in a day for 25 days continuously. The two constituents of the cholinergic system viz. acetylcholine and acetylcholinesterase were determined in selected regions of rat brain such as cerebral cortex, hippocampus, hypothalamus, cerebellum, and pons-medulla at selected time intervals/days under acute and chronic treatment with aluminium. The results revealed that while acetylcholinesterase activity was inhibited, acetylcholine level was elevated differentially in all the above mentioned areas of brain under aluminium toxicity, exhibiting area-specific response. All these changes in the cholinergic system were subsequently manifested in the behavior of rat exhibiting the symptoms such as adipsia, aphagia, hypokinesia, fatigue, seizures, etc. Restoration of the cholinergic system and overt behavior of rat to the near normal levels under chronic treatment indicated the onset of either detoxification mechanisms or development of tolerance to aluminium toxicity in the animal which was not probably so efficient under acute treatment.

## INTRODUCTION

Aluminium, the third most common element in the world, is widely dispersed in abundance in igneous rocks, shales, clays, etc. and by virtue of its greatest properties like strength and corrosion resistance, it has an ever increasing number of applications, ranging from structural materials to thin packaging foils and electrical transmission appliances. Though dietary aluminium is ubiquitous, in small quantities it is not a significant source of concern in persons with normal elimination capacity. However, prolonged exposure causes increased mortality[1] in mice and degeneration of astrocytes,[2] interfering with the metabolism of the neuronal cytoskeleton,[3] encephalopathy in dialysis patients,[4] and implicated in a series of neurological diseases such as amylotrophic lateral sclerosis, dementia associated with Parkinson’s disease, etc.[5]

In view of the above findings, in the present study an attempt has been made to evaluate the toxic effects of aluminium on the cholinergic system in rat brain with reference to its overt behavior, because the cholinergic system is associated with a number of neurological diseases such as epilepsy, dementia, cerebral ischemia, hypoxia, neurodegenerative disorders, etc.[6]

## MATERIALS AND METHODS

Male albino rats, *Rattus norvegicus*, weighing 130 ± 2 gm, of 60 ± 2 days age obtained from Sri Venkateswara Enterprises, Bangalore, were selected as experimental animals and aluminium acetate as the toxicant. The rats were fed with food pellets (Sri Venkateswara Enterprises, Bangalore) and drinking water *ad libitum*. The animals were housed in polypropylene cages under hygienic conditions with photoperiod of 12 hours light and 12 hours dark.

### Parameters studied

#### Toxicity evaluation

Probit method of Finney.[7]

#### Constituents of cholinergic system:

- Acetylcholine : Metcalf method[8] as given by Augustinsson.[9]
- Acetylcholinesterase : Ellman *et al*.[10]

The above biochemical estimations were done under both acute and chronic exposures. For acute exposures, the animals were sacrificed at 1, 3, 6, 12, and 24 hours intervals after oral administration of a single lethal dose of aluminium acetate and for chronic exposures, the animals were treated with sublethal doses of aluminium acetate every days up to 25 day and sacrificed on 5^th^, 10^th^, 15^th^, 20^th^, and 25^th^ day. After cervical dislocation, the brain was isolated quickly and placed in ice. Different areas of the brain such as Cerebral cortex (Cc), Hippocampus (Hc), Hypothalamus (Ht), Cerebellum (Cb), and Pons-medulla (Pm) were isolated by following standard anatomical marks[11] and were immediately homogenized in suitable media for biochemical analysis. The results obtained were analyzed statistically by following standard methods.

#### Behavioral studies

As a corollary to the above, behavioral changes manifested in rat subjected to both acute and chronic doses of aluminium were recorded to coincide with the time intervals selected for cholinergic system.

## RESULTS

**Figure 1:**
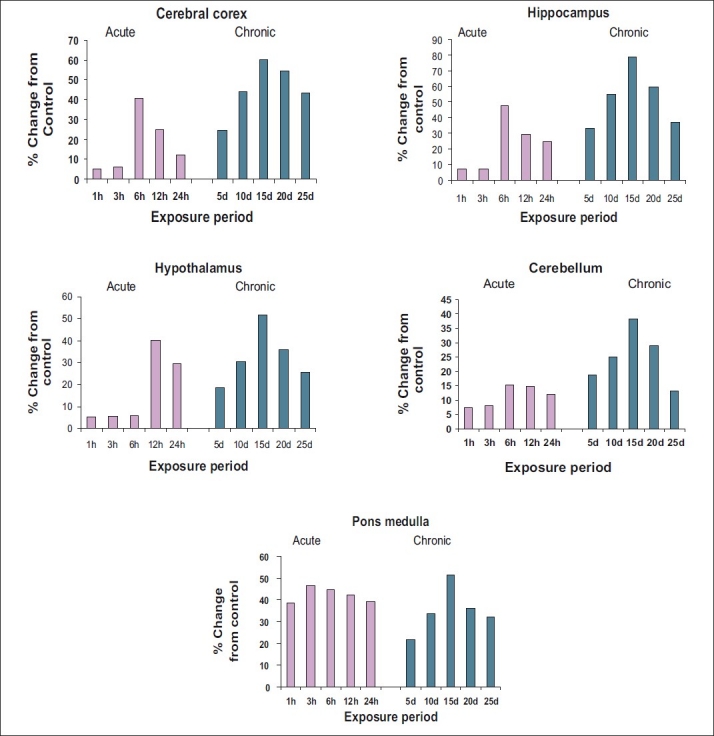
Per cent change in *in vivo* content of acetylcholine in various regions of rat brain following exposure to acute and chronic doses of aluminium acetate

*Acetylcholine:* The results of the present study [Figure 1 and Table 1] clearly indicated that aluminium acetate has significantly altered the level of acetylcholine in all areas of rat brain viz. Cc, Hc, Ht, Cb, and Pm under both acute and chronic exposures. The ACh content in control rat brain was highest in Ht (20.32), followed by CC (18.97), Hc (16.08), Pm (14.22), and Cb (14.10). Under acute doses of aluminium acetate, acetylcholine registered significant elevation in all brain areas of rat at different time intervals in the following order:

**Table 1:** Changes in ACh content (μ moles of ACh/gm) in CC, Hc, Ht, Cb, and Pm of male albino rats exposed to acute and chronic doses of aluminium acetate

	Acute										Chronic									
	C	1h	C	3h	C	6h	C	12h	C	24h	C	5d	C	10d	C	15d	C	20d	C	25d
CC	18.97 ± 0.80	19.98 ± 0.80	18.22 ± 0.9	19.34 ± 0.9	18.25 ± 0.9	25.69 ± 0.9	18.36 ± 0.8	22.98 ± 0.8	18.65 ± 0.9	20.96 ± 0.8	18.90 ± 0.8	23.52 ± 0.7	18.80 ± 0.8	27.09 ± 0.8	18.71 ± 0.9	29.99 ± 0.9	18.53 ± 0.9	28.67 ± 0.9	18.66 ± 0.6	26.77 ± 0.7
		(5.32)*		(6.15)*		(40.76)		(25.16)		(12.39)		(24.44)		(44.10)		(60.29)		(54.72)		(43.46%)
Hc	16.08 ± 0.9	17.17 ± 0.8	16.16 ± 0.9	17.31 ± 0.9	16.28 ± 0.9	24.00 ± 0.9	16.65 ± 0.9	21.58 ± 0.9	16.43 ± 0.8	20.49 ± 0.7	16.39 ± 0.8	21.83 ± 0.8	16.70 ± 0.9	25.87 ± 0.8	16.66 ± 0.6	29.83 ± 0.7	16.82 ± 0.6	26.83 ± 0.8	16.06 ± 0.7	21.99 ± 0.8
		(6.78)*		(7.12)*		(47.42)		(29.61)		(24.71)		(33.19)		(54.91)		(79.05)		(59.51)		(36.92%)
Ht	20.32 ± 0.8	21.34 ± 0.8	20.22 ± 0.9	21.37 ± 0.9	20.00 ± 0.9	21.15 ± 0.9	20.45 ± 0.9	28.69 ± 0.9	20.56 ± 0.9	26.62 ± 0.9	20.78 ± 0.7	24.66 ± 0.7	20.67 ± 0.8	26.97 ± 0.7	20.65 ± 0.9	31.31 ± 0.8	20.44 ± 0.8	27.77 ± 0.7	20.22 ± 0.8	25.44 ± 0.9
		(5.02)*		(5.69)*		(5.75)*		(40.29)		(29.47)		(18.67)		(30.48)		(51.62)		(35.86)		(25.82)
Cb	14.10 ± 0.80	15.15 ± 0.9	14.09 ± 0.9	15.24 ± 0.9	14.07 ± 0.09	16.23 ± 0.9	14.05 ± 0.9	16.12 ± 0.9	14.22 ± 0.8	15.95 ± 0.8	14.3 ± 0.7	16.99 ± 0.7	14.23 ± 0.9	17.79 ± 0.9	14.24 ± 0.6	19.67 ± 0.6	14.13 ± 0.6	18.21 ± 0.7	18.35 ± 0.6	20.77 ± 0.6
		(7.45)*		(8.16)*		(15.35)		(14.73)		(12.17)		(18.81)		(25.02)		(38.13)		(28.87)		(13.19)
Pm	14.22 ± 0.8	19.67 ± 0.8	14.11 ± 0.8	20.68 ± 0.8	14.23 ± 0.9	20.56 ± 0.9	14.44 ± 0.9	20.55 ± 0.9	14.36 ± 0.8	20.01 ± 0.7	14.56 ± 0.6	17.72 ± 0.7	14.38 ± 0.7	19.21 ± 0.7	14.43 ± 0.6	21.88 ± 0.8	14.61 ± 0.7	19.88 ± 0.9	14.32 ± 0.7	18.89 ± 0.7
		(38.33)		(46.56)		(44.48)		(42.31)		(39.35)		(21.70)		(33.59)		(51.62)		(36.07)		(31.91)

Values are mean ± SD of six observations each from tissues pooled from six animals; Values are significant at *P*<0.01; Values in parentheses indicate per cent changes from control; ^ Indicate significance at *P*<0.05;

* Not significant; ACh - acetylcholine; CC - cerebral cortex; Hc - hippocampus, Ht - hypothalamus; Cb - cerebellum; Pm - pons-medulla

Hippocampus > Ponsmedulla > Cerebral cortex> Hypothalamus > Cerebellum (47.42% at 6 hours, 46.56% at 3 hours, 40.76% at 6 hours, 40.29% at 12 hours, 15.35% at 6 hours, respectively)

Similarly, under chronic treatment also, all brain areas showed significant elevation in acetylcholine on 15^th^ day, where Hc recorded maximum elevation (79.05%) followed by CC (60.29%), Pm (51.62%), Ht (51.62%), and Cb the least (38.13%).

### Acetylcholinesterase

**Figure 2:**
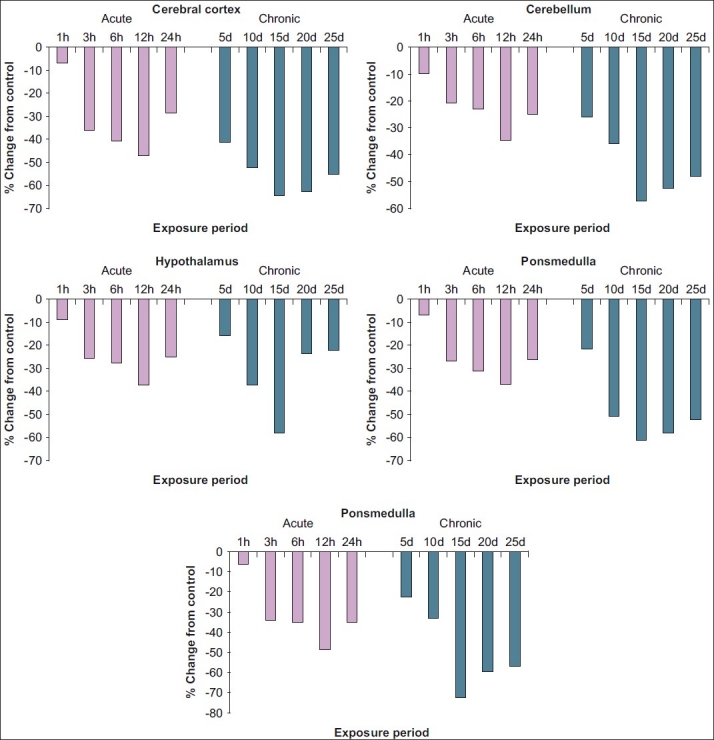
Per cent change in *in vivo* content of acetylcholinesterase in various regions of rat brain following exposure to acute and chronic doses of aluminium acetate

Contrary to acetylcholine, acetylcholinesterase activity [Figure 2 and Table 2] was inhibited by aluminium. The AChE level in various regions of control rat brain was highest in Hc (19.11), followed by Pm (17.19), CC (17.09), Ht (14.20), and Cb (12.28). Under acute exposure, maximum inhibition in acetylcholinesterase was recorded in Hc (46.74%), followed by CC (46.62%), Ht (36.70%), Pm (36.65%), and Cb (33.95%). Under chronic exposure also, a similar trend was noticed with highest inhibition in Hc (69.81%) and least in Cb (56.02%). On comparison, it is obvious that the changes in acetylcholinesterase were more pronounced in rats exposed to chronic doses than acute doses. However, acetylcholine and acetylcholinesterase exhibited recovery tendency from 24 hours onwards in case of acute treatment and from 20^th^ day onwards in chronic exposures.

**Table 2:** Changes in acetylcholinesterase activity (μ moles of ACh hydrolyzed/mg protein/h) in CC, Hc, Ht, Cb, and Pm of male albino rats exposed to acute and chronic doses of aluminium acetate

	Acute										Chronic									
	C	1h	C	3h	C	6h	C	12h	C	24h	C	5d	C	10d	C	15d	C	20d	C	25d
CC	17.09 ± 0.9	16.05 ± 0.91	17.06 ± 0.66	10.88 ± 0.67	16.78 ± 0.34	10.00 ± 0.23	16.88 ± 0.88	9.01 ± 0.80	17.05 ± 0.64	12.25 ± 0.33	17.03 ± 0.18	10.04 ± 0.24	16.99 ± 0.66	8.08 ± 0.67	16.98 ± 0.37	6.02 ± 0.26	16.99 ± 0.28	6.3 ± 0.33	16.99 ± 0.67	7.66 ± 0.54
		(−6.48)*		(−36.23)		(−40.41)		(−46.62)		(−28.15)		(−41.05)		(−52.44)		(−64.54)		(−62.92)		(−54.91)
Hc	19.11 ± 0.92	17.97 ± 0.91	19.36 ± 0.54	12.97 ± 0.65	19.35 ± 0.36	12.80 ± 0.44	19.30 ± 0.67	10.28 ± 0.72	19.36 ± 0.33	12.78 ± 0.47	19.15 ± 0.26	15.05 ± 0.36	19.10 ± 0.52	13.07 ± 0.41	19.08 ± 0.52	5.76 ± 0.41	19.09 ± 0.26	8.08 ± 0.26	19.36 ± 0.61	8.66 ± 0.63
		(−5.97)*		(-33.01)		(−33.85)		(−46.74)		(−33.99)		(−21.40)		(−31.57)		(−69.81)		(−57.67)		(−55. 27)
Ht	14.20 ± 0.93	13.05 ± 0.90	14.42 ± 0.32	10.8 ± 0.21	14.30 ± 0.23	10.34 ± 0.26	14.25 ± 0.66	9.02 ± 0.53	14.15 ± 0.36	10.69 ± 0.45	14.26 ± 0.27	12.06 ± 0.39	14.37 ± 0.36	9.08 ± 0.39	14.36 ± 0.28	6.07 ± 0.38	14.17 ± 0.22	10.87 ± 0.33	14.40 ± 0.42	11.28 ± 0.46
		(−8.10)*		(−25.10)		(−27.69)		(−36.70)		(−24.45)		(−15.43)		(−36.81)		(−57.73)		(−23.29)		(−21.67)
Cb	12.28 ± 0.9	11.13 ± 0.92	12.24 ± 0.23	9.8 ± 0.34	12.26 ± 0.43	9.56 ± 0.27	12.25 ± 0.9	8.09 ± 0.91	12.23 ± 0.26	9.22 ± 0.25	12.10 ± 0.22	9.02 ± 0.19	12.15 ± 0.65	7.86 ± 0.44	12.19 ± 0.66	5.36 ± 0.67	12.17 ± 0.25	5.89 ± 0.26	12.25 ± 0.42	6.5 ± 0.51
		(−9.36)*		(−19.93)		(−22.02)		(−33.95)		(−24.61)		(−25.45)		(−35.31)		(−56.02)		(−51.60)		(−46.94)
Pm	17.19 ± 0.9	16.05 ± 0.91	17.01 ± 0.29	12.46 ± 0.6	17.09 ± 0.34	11.91 ± 0.46	17.00 ± 0.64	10.77 ± 0.46	17.09 ± 0.28	12.92 ± 0.38	17.05 ± 0.40	13.41 ± 0.50	17.35 ± 0.38	8.68 ± 0.35	17.26 ± 0.33	6.8 ± 0.47	17.66 ± 0.64	7.49 ± 0.36	17.01 ± 0.51	8.23 ± 0.53
		(−6.63)*		(−26.75)		(−30.31)		(−36.65)		(−24.4)		(−21.35)		(−49.97)		(−60.60)		(−57.59)		(−51.62)

Values are mean ± SD of six observations each from tissues pooled from six animals; Values are significant at *P*<0.01; Values in parentheses indicate per cent changes from control; ^ Indicate significance at *P*<0.05;

* Not significant; CC - cerebral cortex Hc - hippocampus; Ht - hypothalamus; Cb - cerebellum; Pm - pons-medulla

### Behavioral changes

The behavioral changes exhibited by the rat exposed to acute and chronic doses of aluminium coincided with the selected time intervals/days at which the fluctuations in the cholinergic system occurred. These behavioral changes included adipsia (lack of drinking), aphagia (lack of eating), hypokinesia (reduced locomotor activity), fatigue, seizures, lacrymation, salivation, etc.

## DISCUSSION

Our observation in the present study emphasize that aluminium acetate has induced significant and varied levels of elevation in acetylcholine content and inhibition of acetylcholinesterase activity in all regions of rat brain under both modes of exposure. These observations substantiated that aluminium might be affecting various steps in the metabolic pathway of the neurotransmitters through end-product inhibition. ACh is synthesized in a single reaction from the precursors, acetyl CoA and choline, catalyzed by the enzyme choline acetyl transferase. Accumulation of ACh in various regions of rat brain in the present study under acute and chronic exposures to Al denote an increase in acetyl CoA.[12] Similarly, decreased levels of AChE may be due to attachment of aluminium to the SH-groups of the enzyme at the active sites, thus preventing their functions in certain chemical reactions.[13] Because the molecular weight of aluminium is less than 2000, it could pass through the blood-brain barrier easily and could affect the cholinergic system. The earlier research findings on zinc[14] and lead toxicity[15] in rats lend support to our present findings.

Historically, high levels of Al have not engendered concern because it appears that little of ingested aluminium is absorbed and what is absorbed is rapidly excreted. The absorption of aluminium is poorly understood that both soluble and mucosally associated aluminium metal binding ligands may regulate the initial uptake.[16]

Aluminium toxicity has been recognized in many settings where exposure is heavy and prolonged and renal function is limited. In patients with osteomalacia, there has been a closely associated dialysis encephalopathy caused by aluminium deposition in brain.[2] Because the elimination half-life of aluminium from the human brain is seven years, this can result in cumulative damage of the neurons by interfering with neurofilament axonal transport system, eventually leading to Alzheimer’s-like neurofibrillary tangles. The pathogenesis of aluminium toxicity is complex and may be related to other factors such as impaired parathyroid function and osteomalacia.[17] Excess aluminium is known to exert direct effect on hematopoiesis, poor immunologic response, physical abnormalities such as stuttering, gait disturbance, myoclonic jerks, seizures, coma, abnormal EEG, and sudden death.

Variable levels of elevation in the cholinergic neurotransmitters and its associated enzymes in different brain regions of rat in the present study may be due to heterogeneous nature of the brain tissue and different roles assigned to them such as striatum-signs of toxicity, Cb-cognitive functions, Ht-body temperature, thirst emotions, Cb-equilibrium, Pm-respiratory disorders.[18] Aluminium was known to accumulate in all regions of rat brain upon exposure to acute and subacute doses, maximum accumulation in Hc. Furthermore, aluminium was also seen to compartmentalize in almost all tissues of the body to varying extent, the spleen registering the highest levels.[19] Canned soft drink fed rats had registered significantly higher blood, liver, bone aluminium concentration.[20] The areas of rat brain exhibiting changes in cholinergic system are shown to exhibit the greatest changes in noncholinergic system,[21] thus indicating their possible interdependence. Thus, it is conceived that the adaptive changes underlying tolerance to anticholinesterase agents also involve alterations in other neurotransmitter system in balance with the cholinergic system. Recovery tendency noticed in cholinergic system in all brain regions of intoxicated rat and its behavior further indicate the operation of the detoxification mechanisms and development of behavioral tolerance.

From our present observations, it was obvious that the fluctuations in the cholinergic system under aluminium toxicity coincided well with the frequency and magnitude of the signs and symptoms of several behavioral changes such as adipsia, aphagia, hypokinesia, etc. These observations further support that aluminium might have caused lesions in the important regions of brain like substantia nigra, Ht, etc. These motor deficits and motivational changes are closely associated with some of the symptoms characteristic to Parkinson’s disease.[22] It has been reported that three patients who worked in aluminium smelting industry for 12 years were presented with severe asthma[23] and a progressive neurological disorder viz. Potroom palsy with uncoordinated movements, tremors, cognitive deficits, etc.[24] Furthermore, there is ample evidence that neurotoxic effects of aluminium in animals are directed at the central nervous system and long-term and low-level exposure to aluminium could explain the reasons for the Potroom palsy. These earlier reports on the aluminium toxicity to nervous system and finally culminating in development of neurodegenerative disorders[25] in human beings altered its status from being a nontoxic, nonabsorbable, and harmless element to highly toxic heavy metal.

Regardless of the host and the route of administration, aluminium is proved as a potent neurotoxicant. In the young, adult, or developmentally matured host, the neuronal response to aluminium exposure can be dichotomized on morphological grounds, one involving intraneuronal neurofilamentous aggregation and the other producing significant neurochemical and neurophysiological perturbations such as speech disturbances and abnormal EEG, progressive encephalopathy with muscular atrophy, reduced mental development index, etc.[26] Aluminium toxicity is a wide-spread problem in all forms of life including human beings, animals, fishes, plants, and trees and causes wide spread degradation of environment and death. Even though aluminium is not considered to be a heavy metal like lead, it can be toxic in excessive amounts and even in small amount, if deposited in brain.

All our observations in the present study provide conclusive evidences that the aspect of aluminium toxicity to human beings needs special attention from the environmentalist point of view because it might increase the risk of occupational hazards with particular reference to neurological diseases.
